# Using Human Movement Data to Identify Potential Areas of Zika Transmission: Case Study of the Largest Zika Cluster in Singapore

**DOI:** 10.3390/ijerph16050808

**Published:** 2019-03-05

**Authors:** Jayanthi Rajarethinam, Janet Ong, Shi-Hui Lim, Yu-Heng Tay, Wacha Bounliphone, Chee-Seng Chong, Grace Yap, Lee-Ching Ng

**Affiliations:** 1 Environmental Health Institute, National Environment Agency, 11 Biopolis Way, #06-05-08, Singapore 138667, Singapore; jayanthi_rajarethinam@nea.gov.sg (J.R.); janet_ong@nea.gov.sg (J.O.); wacha.bounliphone@gmail.com (W.B.); chong_chee_seng@nea.gov.sg (C.-S.C.); 2 Starhub Limited, 67 Ubi Avenue 1, #05-01 StarHub Green, Singapore 408942, Singapore; shihui713@gmail.com (S.-H.L.); tayyh89@gmail.com (Y.-H.T.); 3 Environmental Public Health Operations, National Environment Agency, 40 Scotts Road, #13-00 Environment Building, Singapore 228231, Singapore; grace_yap@nea.gov.sg; 4 School of Biological Sciences, Nanyang Technological University, 60 Nanyang Drive, Singapore 637551, Singapore

**Keywords:** zika, human movement, mobile phone data, disease transmission, risk

## Abstract

Singapore experienced its first Zika virus (ZIKV) cluster in August 2016. To understand the implication of human movement on disease spread, a retrospective study was conducted using aggregated and anonymized mobile phone data to examine movement from the cluster to identify areas of possible transmission. An origin–destination model was developed based on the movement of three groups of individuals: (i) construction workers, (ii) residents and (iii) visitors out of the cluster locality to other parts of the island. The odds ratio of ZIKV cases in a hexagon visited by an individual from the cluster, independent of the group of individuals, is 3.20 (95% CI: 2.65–3.87, *p*-value < 0.05), reflecting a higher count of ZIKV cases when there is a movement into a hexagon from the cluster locality. A comparison of independent ROC curves tested the statistical significance of the difference between the areas under the curves of the three groups of individuals. Visitors (difference in AUC = 0.119) and residents (difference in AUC = 0.124) have a significantly larger difference in area under the curve compared to the construction workers (*p*-value < 0.05). This study supports the proof of concept of using mobile phone data to approximate population movement, thus identifying areas at risk of disease transmission.

## 1. Introduction

The emergence of dengue (DENV), chikungunya (CHIKV) and zika (ZIKV) [1,2,3,4,5] viruses into new susceptible human populations, driven in part by human travel on both local and global scales, represents significant public health concerns, particularly in Singapore with suitable climatic conditions and constant urbanization. Unlike CHIKV and DENV, ZIKV’s association with Guillain–Barré syndrome and microcephaly cases was a major cause of public alarm during the epidemics [6]. In addition, a study has reported that the social and economic impacts of the recent Zika outbreak in Latin America and the Caribbean alone could cost countries in the region of an estimated 7–18 billion dollars from 2015 to 2017 respectively [7]. Reports in 2016 confirmed the introduction and spread of ZIKV in Southeast Asia and that the likelihood of under-diagnosis is high [8]. It is imperative to have timely health care response and outbreak control measures, which are focused in areas predicted to be at the highest risk of transmission, to effectively control outbreaks of new infectious diseases [9].

Human movement is known to play an important role in the transmission dynamics of diseases [10,11,12]. With the recent emergence of mobile phone calling data and associated location information, experts have suggested using mobile phone data to understand the spatial and temporal spread of disease incidences [13]. One such study was recently conducted on the 2010 Cholera epidemic in Haiti, whereby anonymized mobile data enabled the prediction of epidemic spread [14]. In addition, another study on the same epidemic also showed that it was feasible to monitor real-time population movements using mobile phone data during infectious disease outbreaks, thus allowing for better preparedness in controlling outbreaks [15]. Studies conducted by Amy Wesolowski et al. have shown the possibility of using mobile phone–based mobility estimates to predict the geographic spread of vector-borne diseases, such as dengue epidemics as well as malaria prevalence [16,17].

In August 2016, Singapore experienced its first local zika outbreak [5,18]. When the first locally acquired ZIKV case in Singapore was identified, the Ministry of Health (MOH) conducted active case findings for the purpose of outbreak investigations and public health measures [19,20]. The National Environment Agency (NEA) intensified its battle against the *Aedes* mosquitoes to interrupt transmission. In addition to vector control measures, public outreach was stepped up to urge residents to clear potential breeding habitats to prevent mosquito breeding.

As ZIKV was newly introduced in Singapore, this outbreak provided an opportunity to understand the implication of human movement on disease spread. A total of 460 ZIKV cases were reported in Singapore in 2016, among which 64.8% (*n* = 298) belonged to the first ZIKV cluster, known as the Aljunied Crescent cluster. We postulated that human movement from the Aljunied Crescent cluster locality led to the spread of ZIKV and that different segments of population contributed differently to the spread. Therefore, a retrospective study was conducted to determine how the movement of different groups of individuals (construction workers, residents, visitors) from the Aljunied Crescent cluster locality to other localities influenced ZIKV transmission. We used reported cases of ZIKV infection to validate the accuracy of the identified areas. The results of this study would enable the identification of risk areas of disease transmission areas for targeted intervention. The key aim of this study is to see if only human movement data is sufficient to accurately predict the spread of the disease. Such a predictive tool could help public health officers prioritise areas for investigation, inspections and vector control, to preemptively mitigate the risk of transmission.

## 2. Materials and Methods

### 2.1. Data Collection

ZIKV case data. In Singapore, the MOH is responsible for the epidemiological and clinical management of ZIKV, while NEA is responsible for the vector surveillance and control. A ZIKV case is defined by a person who is tested positive for confirmed ZIKV in serum or urine samples by the reverse transcription polymerase chain reaction method. All cases are notified to MOH and epidemiological investigations are then conducted via interviews to obtain epidemiological data. Epidemiological information such as residential addresses and dates of onset of illness and diagnosis of patients were collected for this study. The permission to use these data was approved by MOH, in Singapore on 5 July 2018. All laboratory-confirmed and locally-acquired ZIKV cases reported to MOH in 2016 were anonymized and aggregated to hexagons prior to analysis.

ZIKV cluster. In Singapore’s *Aedes* borne disease control programme, a ZIKV cluster of two cases located within 150 m from each other and with onset dates within 14 days [5], suggests a localized transmission. Subsequent cases that fulfill the same criteria are also tagged to the same cluster. Using these criteria, the Aljunied Crescent cluster was considered active from 29 August 2016. As all locally acquired ZIKV cases were used in the analyses, the assumption we made was that all the cases were epidemiologically linked to one another. Hence, we assumed that the spread of the ZIKA transmission around the island started off from cases at the Aljunied Crescent cluster locality.

**Figure 1:**
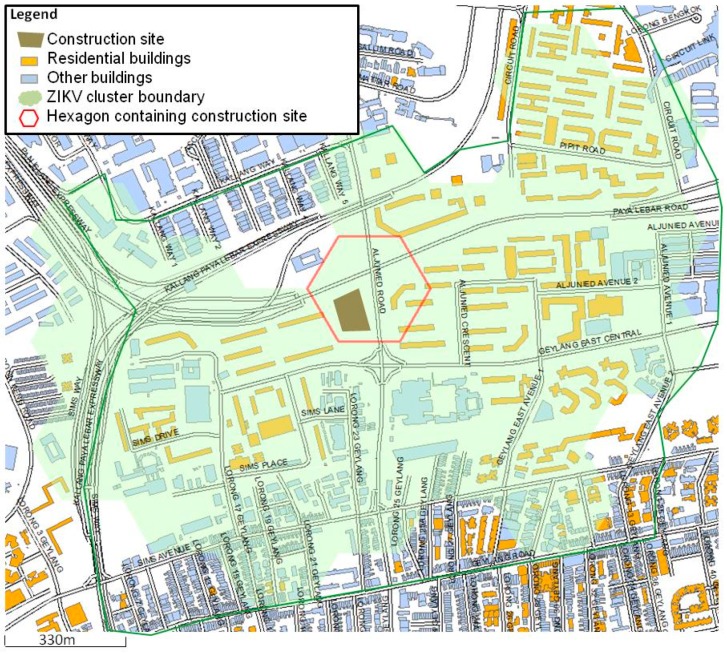
Study area. The shaded green area is the Aljunied crescent ZIKV cluster boundary. The hexagon highlighted in red contains the implicated construction site. The figure was created using ArcGIS version 10.5.

Study area. Singapore is divided into regular hexagons each with a circum-radius of 165 m and an average area of 0.07193 km^2^. There is a total of 11,401 hexagons, out of which 5,176 (45.4%) are residential hexagons. The Aljunied Crescent ZIKV cluster locality, which is defined as the area where the ZIKV clustered cases are reported, consists of 24 hexagons, with a total area of 1.72 km^2^ (Figure 1). The Aljunied Crescent ZIKV cluster locality comprises an approximate human population size of 34,000. The area is a mixture of residential buildings; commercial buildings such as shop-houses; and other amenities like schools, swimming complex, hospitals as well as a train station. During the period of this analysis, there was an ongoing residential construction site located within the cluster locality. The Aljunied-Kallang neighbourhood, where the cluster occurred, has a historical presence of *Ae. aegypti* and was categorised as a high-risk area for dengue transmission [5].

Mobile phone data. Call data records (CDRs) provide a detailed temporal and spatial movement of subscribers of mobile telephone companies (Telcos). The present study was based on all CDRs from three one-week periods from 13–19 June 2016, 25–31 July 2016 and 8–14 August 2016. This period was selected because the transmission period of the ZIKV cluster was from 31 July 2016 to 25 September 2016. Hence, the period selected for this analysis starts from 2 months prior to the onset of the cluster, as well as during peak transmission periods of the cluster. The data contained a total of 70,147 subscribers (for the three weeks) of StarHub limited (Ltd), one of the three major Telcos in Singapore, which constituted approximately 30% of the coverage of total mobile subscribers in Singapore. Aggregated Telco insights are availed through data products developed on anonymized data, respecting consumer privacy and strict adherence to personal data protection legislation. The network is represented by nodes and entities, where nodes are the entities and links are the relationships between the entities. In this study, the nodes refer to the regular hexagons of size 165 m, and the links refer to the connectivity between the pairwise hexagons (i.e., number of subscribers travelling from one hexagon to another). GRID360, a geo-location product developed by StarHub Ltd. to anonymize and aggregate island-wide geolocation-related data onto regular hexagons of size 165 m, was deployed by StarHub Ltd. to detect dwelling points of individual subscribers and subsequently their origin and destination locations of travel by analyzing their cell phones’ network activities. The GRID360 origin–destination location engine detects a dwelling point (origin or destination) when the subscriber has stayed in a hexagon for 30 min or longer. Each unique subscriber in our data corresponds to a single geographical location and is generated when the following occur:

1. Whenever there are activities on the phone which engage a service of the Telco, such as usage of mobile data, including applications running in the background, calls or short message services.
2. Whenever the phone moves into another location area code.
3. Periodically, there is a location update (roughly once every 2 to 3 h) if there is no activity or movement to another location area code.

### 2.2. Quantifying Human Movement Using Mobile Phone Data

Firstly, human movement from Aljunied Crescent ZIKV cluster locality was developed. The Aljunied Crescent ZIKV cluster locality was the origin. All other hexagons with movements into them were destinations. Human movement data was categorized into three groups of individuals: (1) construction workers, (2) residents and (3) visitors.

1. Construction workers: Subscribers who dwelled in the construction site hexagon within the Aljunied Crescent ZIKV cluster locality at least five times per week for more than 4 h on each occurrence, were at least 18 years old and were from the following countries (Malaysia, People’s Republic of China, India, Sri Lanka, Thailand, Bangladesh, Myanmar, Phillipines, Hongkong, Macau, South Korea and Taiwan) and categorized as construction workers. The countries were chosen as per the Ministry of Manpower’s work permit requirement criteria for construction workers [21].
2. Residents: Subscribers who dwelled in the Aljunied Crescent ZIKV cluster locality at least five times per week for more than 4 h on each occurrence and resided in the locality (based on their home addresses) were categorized as residents. There will be no overlap with the category of construction workers, as construction workers do not have residential addresses within the area.
3. Visitors: subscribers who dwelled in the Aljunied Crescent ZIKV cluster locality at least five times per week for more than 4 h on each occurrence and did not reside in the locality (based on their home addresses) were categorized as visitors. In this category also, subscribers who overlapped with the category of construction workers were excluded from this group.

Analyses of the retrieved Telco data using a chi-square test showed that the data was comparable to Singapore’s population data for each of the variables: age group, gender and ethnicity. Hence, we assume that the data is representative of the whole population.

### 2.3. Statistical Analyses

The odds ratio (OR) was calculated to find out the odds of ZIKV cases reported in a hexagon that had human movement from the cluster locality into a hexagon, compared to the odds of ZIKV cases reported in a hexagon in the absence of human movement from the cluster locality into a hexagon. OR was calculated for total subscribers included in the study, as well as individually for different group types. A chi-square test for independence was used to test for significance between the variables. OR was calculated using the Formula below:

OR = (a/c)/(b/d)

where:

“a” denotes the number of hexagons reported with at least one ZIKV case and at least one person moving into a hexagon.
“b” denotes the number of hexagons with no ZIKV case and at least one person moving into a hexagon.
“c” denotes the number of hexagons with at least one ZIKV case and nobody moving into a hexagon.
“d” denotes the number of hexagons with no ZIKV case and nobody moving into a hexagon.

The Receiver Operating Characteristic (ROC) curve in this analysis depicts the sensitivity (true positive rate) and specificity (true negative rate) of using origin–destination location insights to predict the ZIKV occurrences in hexagons. Sensitivity refers to the proportion of hexagons that had movement and ZIKV cases (true positive rate); and specificity refers to the proportion of hexagons that had no movement and no ZIKV cases (true negative rate). False positive rate refers to the hexagons that were identified as having ZIKV cases although they did not. False negative rate refers to the hexagons that were identified as not having ZIKV cases although they had. ROC curves were generated for each of the three groups of individuals. The AUC of the ROC curves provides a measure of how well the human movement of the particular group is able to predict the occurrence of the ZIKV cases in the hexagons. The ROC curves of the three groups were independent of each other. Hence, a comparison of independent ROC curves was used to test the statistical significance of the difference between the areas under the ROC curves of the three groups of individuals (comparing ROC curve of residents with ROC curve of visitors, comparing the ROC curve of residents with the ROC curve of construction workers and comparing the ROC curve of visitors with the ROC curve of construction workers) with the non-parametric method described by Delong et al. [22].

The value of AUC using the empirical method is calculated by summing the area of the trapezoids that are formed below the connected points making up the ROC curve. From DeLong et al. [23], we define the T1 component of the *i*th subject, *V*(*T*_1i_) as

$$V\left(T_{1i}\right)=\;\frac{1}{n_{0}}\overset{n_{0}}{\underset{j=1}{\sum }}¥\left(T_{1i},T_{0j}\right)$$

And define the *T*_0_ component of the jth subject, *V(T*_0j_) as

$$V\left(T_{0j}\right)=\;\frac{1}{n_{1}}\overset{n_{1}}{\underset{i=1}{\sum }}¥\left(T_{1i},T_{0j}\right)$$

where

¥ (X,Y)= 0 if Y>X,¥ (X,Y)= 12 if Y=X,¥ (X,Y)= 1 if Y<X

The empirical AUC is estimated as

$$A_{Emp}=\overset{n_{1}}{\underset{i=1}{\sum }}V(T_{1i})/n_{1}=\overset{n_{0}}{\underset{j=1}{\sum }}V\left(T_{0j}\right)/n_{0}$$

The variance of the estimated AUC is estimated as

$$V\left(A_{Emp}\right)=\frac{1}{n_{1}}S_{T_{1}}^{2}+\frac{1}{n_{0}}S_{T_{0}}^{2}$$

where $S_{T_{1}}^{2}$ and $S_{T_{0}}^{2}$ are the variances

$$S_{T_{1}}^{2}=\frac{1}{n_{j=1}}\overset{n_{i}}{\underset{i=1}{\sum }}{\left[V(T_{1i})-A_{Emp}\right]}^{2},i=0,1$$

Spatial maps were created in ArcGIS version 10.5 (Environmental Systems Research Institute, Inc. (ESRI, Redlands, CA, USA). R software version 3.0.2 (R: A language for environment and statistical computing, Vienna Austria) was used for statistical tests. Statistical significance was assessed at the 5% level.

## 3. Results

**Figure 2:**
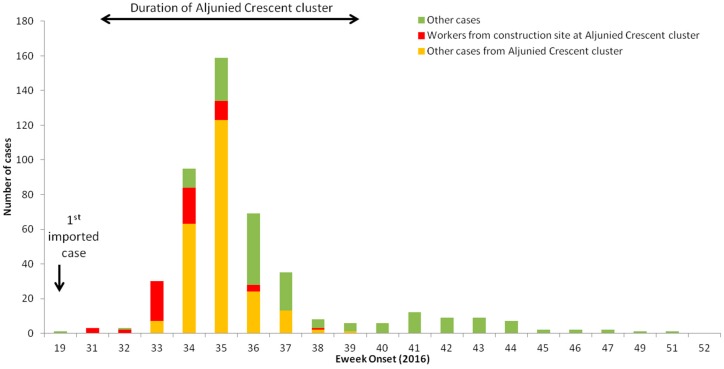
Weekly number of reported ZIKV cases in 2016. Cases in Aljunied cluster from August–October 2016 highlighted in red and orange; cases working in the adjacent construction site are red and the rest of the cases in the cluster are orange.

A total of 460 ZIKV cases were reported, among which 64.8% (*n* = 298) belonged to the Aljunied Crescent cluster. The majority of the initial cases (80% of the 1st three weeks of onset of the illness, epidemiological week 31–33) reported in the cluster were workers from the construction site (Figure 2). 

**Figure 3:**
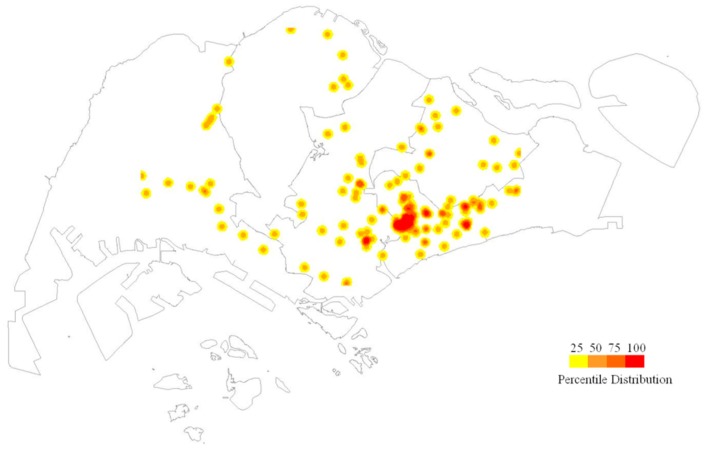
Spatial distribution of Zika cases in 2016. The spatial distribution of Zika cases in Singapore was generated using the kernel density tool in the spatial analyst toolbox of ArcGIS 10.5 ArcMap software (ESRI, CA, USA) based on a search radius of 400 m. Case density values were classified into four classes of <25th (2 cases/km^2^), 25th–50th percentile (3–8 cases/km^2^), 51st–75th percentile (9–29 cases/km^2^) and more than 75th percentile (>29 cases/km^2^) quantiles, using the quantile classification method and were displayed in tones of yellow, orange and red respectively, as shown in the legend.

The ZIKV density map showed that though cases were concentrated in the eastern side of Singapore, we also see sporadic cases spread around the island (Figure 3).

**Figure 4:**
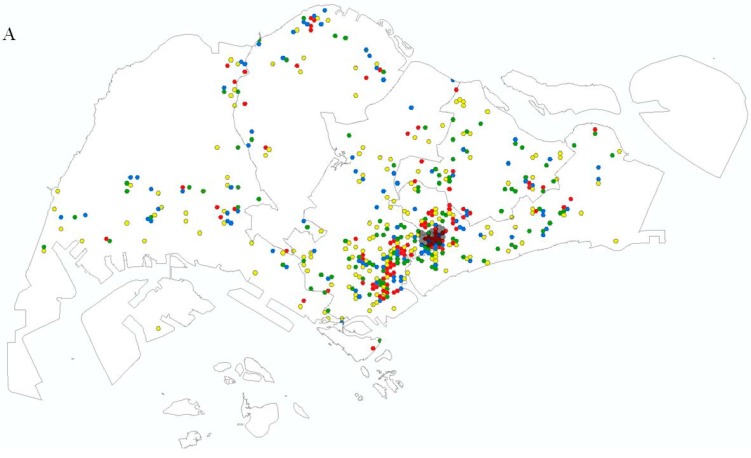

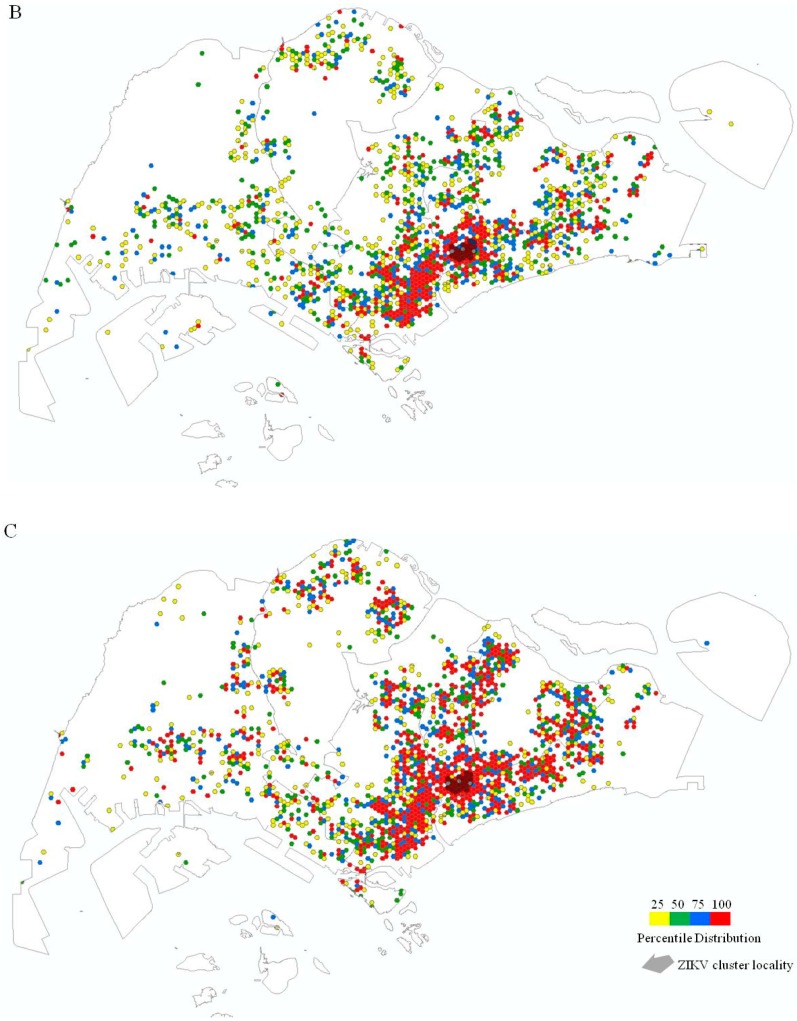
Maps (**A**–**C**) show the movement of construction workers, residents and visitors from the Aljunied Crescent ZIKV cluster, respectively. The number of subscribers that move into the residential hexagons from the Aljunied Crescent ZIKV cluster were classified into four classes of <25th (1 subscriber) in yellow, 25th–50th (2–3 subscribers) in green, 51st–75th (4–8 subscribers) in blue and more than 75th (9 and above subscribers) in red using the quartile classification method. Five-thousand-one-hundred-and-seventy-six residential hexagons are overlaid on the map of Singapore. A similar movement pattern was observed for the three periods that were selected. The Aljunied Crescent ZIKV cluster locality is overlaid on the map. The figure was created using ArcGIS version 10.5.

There were a total of 5176 residential hexagons in Singapore, and the 460 ZIKV cases were reported in 173 hexagons. Out of these, 24 hexagons belonged to the Aljunied Crescent Locality. The odds ratio of ZIKV cases in a hexagon visited by a subscriber from the Aljunied Crescent cluster locality, independent of the group of individuals, is 3.20, reflecting a higher count of ZIKV cases when there is a movement into a hexagon from the cluster locality. It is statistically significant, as evidenced by the 95% CI (2.65–3.87) which does not include the value 1 (the null hypothesis is understood to be odds ratio = 1) and a Chi-square test of independence yields a *p*-value < 0.05. Moreover, when we take into consideration the different groups of individuals, the effect of this confounding variable give us similar odds ratios. Table 1 presents the total number of subscribers moving into a hexagon from the cluster locality and the odds ratio of reported ZIKV cases among different groups of individuals. The odds ratio of having ZIKV cases reported in a hexagon visited by a subscriber from the cluster locality for construction workers is 1.15 and 0.3 lower than residents and visitors respectively, as we had anticipated. The reason is because construction workers had limited movement, with at least 20% fewer hexagons visited compared to residents and visitors. In contrast, the odds ratio of ZIKV cases from the cluster locality moving into a hexagon for residents is higher at 4.24. Furthermore, for all groups of individuals, the overall case burden of hexagons visited by at least one person from the Aljunied Crescent cluster locality is significantly higher than that of remaining hexagons, as evidenced by the 95% CI. Figure 4 shows that residents and visitors had a broader range of movement, with a higher density of subscribers found in the southern and south-eastern part of Singapore. The spatial distribution of the subscribers is comparable to the spatial distribution of the population living in Singapore, as shown by the human population density segregated by subzones [23]. In contrast, construction workers had a more limited range of movement.

**Figure 5:**
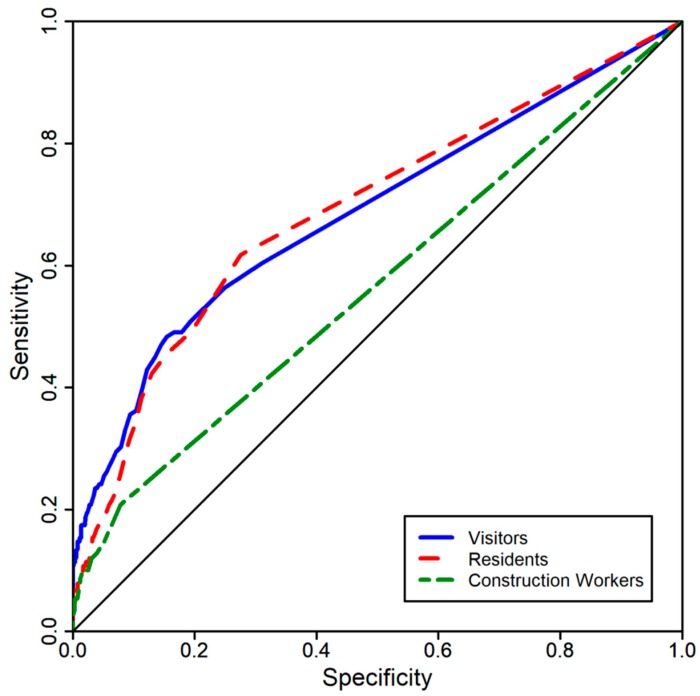
ROC curves (sensitivity and specificity) for predicting the ZIKV occurrences based on the human movement of the three groups of people: blue line denotes visitors, red line denotes residents and green dotted line denotes construction workers. The area under curve (AUC) for visitors, residents and construction workers are 0.686 (0.640–0.732 95% CI), 0.691 (0.647–0.734 95% CI) and 0.567 (0.533–0.600 95% CI), respectively.

Comparison of the independent ROC curves showed that visitors and residents have a significantly larger difference in area under the curve (AUC) compared to the construction workers using the non-parametric method of Delong et al. (*p*-value < 0.05) (Figure 5, Table 2). There is no significant difference in AUC between residents and visitors (*p*-value > 0.05)

## 4. Discussion

This study shows that there are higher odds of ZIKV cases being reported in areas that were visited by people from areas with active ZIKV transmission compared to areas that were not (Table 1). However, the AUC (visitors = 0.686, residents = 0.691, construction workers = 0.567) was moderate for visitors and residents and low for construction workers suggesting that using only human movement is not good enough to predict risk areas with high accuracy. Nevertheless, the findings are particularly important for improving early containment efforts of vector-borne diseases. This finding is supported by several studies that have demonstrated the role of human movement in the spatial spread of diseases [10,11,12,13]. Singapore is highly susceptible to infectious disease outbreaks due to multiple factors: (i) Singapore being a travel hub, (ii) having one of the highest population densities in the world (7796 people per square kilometer) [24] and (iii) improved local transportation that facilitates virus dissemination. Hence, human movement data can be used as a risk assessment tool to identify risk areas to ensure that resources are deployed in a strategic and sustainable manner.

Human movement is not the only risk factor for ZIKV transmission, as it is a vector-borne disease. The data shows that human movement during the daytime is heavily aggregated in south and southeast Singapore. However, we did not have any clusters in many of these locations. For active transmission to be established in the areas visited by the individuals from the cluster locality, other critical factors such as land use, demography, presence of vectors, presence of naïve humans, human density and conducive weather parameters are required. The accuracy of the potential risk areas identified can be improved by overlaying the spatial distribution of *Aedes* population. The technique was explored in a recent study in Namibia, where human movement data together with case data was used to prioritize areas for surveillance and control of Malaria [25,26]. In Singapore, a similar technique is currently used for dengue control. A dengue risk map was developed by modeling multiple layers of data such as past dengue cases, presence of mosquitoes, human population density and environmental factors to guide vector control operations [27].

In Singapore, the range of movement of different segments of population can differ based on their lifestyles. Therefore, we categorized human movement data into different groups of individuals (residents, visitors and construction workers) to find out the impact of each group on the spread of the disease. A past study conducted in the Environmental Health Institute has shown that a construction site is a potential environmental driver for increased dengue transmission, and that the odds of dengue clusters, associated with construction sites expanding into large clusters, are 9 to 17 times higher than clusters not associated with construction sites [28]. This is because large-scale building construction activities often create environmental conditions conducive for vector-borne disease transmission due to the increased likelihood of *Aedes* breeding habitats. Also, construction workers, who are generally from dengue non-endemic countries, are prone to dengue infections. As a result, we postulated that they might be a key factor in facilitating dengue transmission in residential areas that are located in close proximity to construction sites. ROC analysis showed that residents and visitors of the cluster were more likely to cause the spread of ZIKV around the island as compared to the construction workers. This is likely due to the smaller range of movement of construction workers, who tend to stay within their worksites and dormitories. However, it is possible that workers who stayed within their worksites might have been responsible for diffusing ZIKV in the residential areas close to worksites especially at the beginning of the outbreak, which is then spread to other parts of the island. We also noticed that the dormitories were located in areas with low *Ae.aegypti* population, which could be a possible reason why an outbreak was averted [29]. By categorizing human movement based on the population of interest, this study has shown us more insights into the spatial distribution in relation to disease spread which can be important to better understand localized spread of diseases, such as within a community, for targeted control efforts.

One key limitation of this study is in the quantification of human movement based on mobile data. The inference of the different categories in the data is unlikely to be without error. For example, the method to infer the categories was a trade-off in tuning the appropriate hyper parameters to maximize the chances of identifying data that exhibited a category. For instance, construction workers’ behavior is sufficient to believe that they were likely to be workers associated with the construction site in the cluster. Additionally, the data, being dependent on the frequency of mobile phone usage, can vary for different groups. For example, no data will be generated for mobile phones that have been switched off. Another concern is that the data collected by the Telco used in this study, represented approximately one third of the total mobile phone users in the market. Nonetheless, this data is expected to have little impact on the spatial patterns observed in this study.

Another key limitation is that data was collected from three one-weeks periods in June, July and August and not throughout the epidemic transmission. An analysis of CDRs using Kendall’s tau-b correlation of the movement of the general human population has shown that there is no significant difference in the movement data on a month-to-month basis. Therefore, the data is expected to be a sufficient representation of movement data throughout the epidemic transmission.

Another significant limitation is that the association between human movement and ZIKV cases was done as a binary analysis. As the mobile phone data was aggregated to a hexagon level, analyses did not take into account the association between the distance of movement between zones and intensity of ZIKV transmission. More in-depth data should be collected for such analyses. For successful future use of this method in outbreak control of infectious diseases, having access to aggregate data on mobility between areas on a regular basis is necessary to identify risk areas. Hence, it will be ideal to have infrastructure and systems in place for such aggregated connectivity data to be collected from all the Telcos present in Singapore to assist decision making processes for future outbreaks.

## 5. Conclusions

In summary, this study supports the proof of concept of using mobile phone data to approximate population movement, which can be used to identify areas at risk of disease transmission. This method is especially useful for newly introduced human-to-human diseases. By quickly identifying possible areas of transmission, operations can be carried out in a timely manner to help mitigate the spread. Further studies are warranted to include other risk factors such as vector population and herd immunity to improve the accuracy of the risk areas. Despite having a low mosquito population, Singapore continues to be under threat of vector-borne diseases transmission due to several factors such as current dynamics of climate change, globalisation, travel, trade, socioeconomics as well as viral evolution [30]. Hence, the current vector control regime has to be constantly enhanced by incorporating data analytics to be more effective.

## Figures and Tables

**Table 1:** Comparison of the number of subscribers moving into a hexagon from the Aljunied Crescent cluster and the OR of ZIKV cases reported in a hexagon form the Aljunied Crescent cluster for the three groups of individuals.

	Construction Workers	Residents	Visitors
Total number of hexagons with movement into them (%)	425 (8.2%)	1478 (28.6%)	1651 (31.9%)
Odds ratio (95% CI)	3.09 (1.98–4.69)	4.24 (3.00–6.04)	3.39 (2.40–4.81)

**Table 2:** Comparisons of independent ROC curves with the non-parametric method of Delong et al. [22] of the three groups of individuals: construction workers, visitors and residents.

Groups of People	Difference in Area under Curve (AUC)	95% CI	Standard Error
construction workers and residents	0.124 (*p* < 0.05)	0.069–0.179	0.0279
construction workers and visitors	0.119 (*p* < 0.05)	0.062–0.176	0.0291
residents and visitors	0.005	−0.058–0.068	0.0323
